# Machine Learning Classifiers for Voice Health Assessment under Simulated Room Acoustics^†^

**DOI:** 10.3390/engproc2024081016

**Published:** 2025-05-07

**Authors:** Ahmed M. Yousef, Eric J. Hunter

**Affiliations:** 1 Department of Communication Sciences and Disorders, University of Iowa, Iowa City, Iowa 52240, USA;

**Keywords:** room acoustics, voice disorders, machine learning, voice assessment, speech acoustics, reverberation, support vector machine, random forest, overfitting, data augmentation

## Abstract

Machine learning (ML) robustness for voice disorder detection was evaluated using reverberation-augmented recordings, highlighting data quality’s impact. Common vocal health assessment voice features from steady vowel samples (135 pathological, 49 controls) were used to train and test six ML classifiers. Detection performance was evaluated using clean and 2 simulated room reverberation situations (short=0.48s, long=1.82s). Support Vector Machine and k-Nearest Neighbors demonstrated reliable accuracy under short/acceptable reverberation, while Random Forest achieved the highest accuracy on clean data but lacked generalizability in augmented room conditions. Training/testing ML models on augmented data is essential to enhance their reliability in real-world voice assessments.

## 1. Introduction

Using voice and speech signals as a potential key marker of vocal health, machine learning (ML) has emerged as a powerful tool in automating voice assessment and screening for voice disorders [1–3]. These capabilities hold great potential for monitoring treatment progress, tracking patient follow-ups, and improving accessibility to voice care [4,5].

Previous studies have demonstrated the high efficacy of ML models in classifying and identifying those with vocal abnormalities, dysfunctions, and voice disorders, which conditions are often linked to broader health conditions [6–9]. However, most of this research has relied on voice samples collected under highly controlled recording conditions, such as in soundproof rooms with minimal noise and reverberation [10–12], which is not reflective of real-world clinical settings, which often include varied noise and reverberation conditions [13–15]. As a result, the generalizability of these models remains significant limitations due to a lack of understanding of the impact of various room conditions. With increased understanding of the impact, there is an opportunity to use a larger dataset [16,17], diverse voice samples [7,18], model optimization [19,20], and other techniques [21–24] which could significantly expand the widespread adoption of ML in clinical practice [1,2,4,25].

This study addresses this gap by evaluating the accuracy of different ML models in distinguishing voice disorder samples from healthy voice samples where the samples were augmented to represent extreme, common, and low reverberation conditions. By testing these models on samples from such diverse conditions, the study provides valuable insights into identifying robust models and strategies to enhance their generalizability—better reflecting their performance in real-world settings and improving their clinical utility.

## 2. Materials and Methods

### 2.1. Voice Samples under Simulated Room Acoustics

Audio samples from 49 vocally normal individuals and 135 patients with clinically diagnosed voice disorders, including both organic and nonorganic pathologies, were used. Each sample consisted of a sustained vowel /a:/ produced at habitual pitch and loudness and later trimmed to 3 seconds of vowel, a common voice production assessment task [26,27]. These recordings were captured in acoustically treated room using a head mounted microphone with low noise and reverberation, serving as the baseline for the study. All original recordings from the low-reverberation condition (“low reverb”) were also augmented to simulate a common reverberation level of a standard clinic room (0.48 seconds, “med reverb”) and a high reverberation level like in a large church or gymnasium (1.82 seconds, “high reverb”). The overall dataset for training and testing thus consisted of all 184 “low reverb” audio samples and two more versions (“med reverb”, “high reverb”) for a total of 552 samples. The reverberation effects were simulated using the Reverb tool in Audacity (version 2.4.1), an audio editing software [28].

A training subset (80%) was created by randomly selecting “low reverb” samples (named “training recordings”) from both healthy speakers and patients using a stratified technique to ensure equal representation of both groups. The remaining 20% of the “low reverb” recordings were retained as a testing subset for evaluating the ML models. The “low reverb” testing subset recordings were matched with their corresponding “medium reverb” and “high reverb” versions, forming two additional testing subsets. Thus, the three testing subsets contain the identical audio content but differ in audio quality, simulating different room acoustic conditions.

### 2.2. Machine Learning for Voice Disorder Screening

Twenty acoustic voice parameters commonly used in voice quality assessment were extracted from all audio recordings, representing various temporal, spectral, and cepstral features of the acoustic voice signal. These measures were computed using PRAAT, a freely available software for voice and speech analysis [29]. The acoustic metrics generated from the “training recordings” formed a training set used to develop a variety of supervised ML models. These models, implemented using the default Scikit-learn library in Python [30], were designed as binary classifiers to detect voice disorders in the audio samples and classify them as either healthy or pathological. The models included Random Forest (RF), Gradient Boosting (GB), Support Vector Machine (SVM), Extra Trees (ET), AdaBoost (AB), and k-Nearest Neighbors (k-NN).

Three different sets of features were extracted, corresponding to the three testing recording subsets: “low reverb”, “med reverb”, and “high reverb”. These testing features were used to evaluate the classification performance of the models. Each model was evaluated three times on each testing subset to assess its robustness against different levels of challenging audio quality, ranging from clean/regular (low reverberation) to highly reverberant recordings. The classifiers were evaluated using receiver operating characteristic (ROC) curves, Area Under the Curve (AUC), overall classification accuracy, and F-score to compare the impact on ML performance before and after adding reverberation effects.

## 3. Results

**Figure 1.**
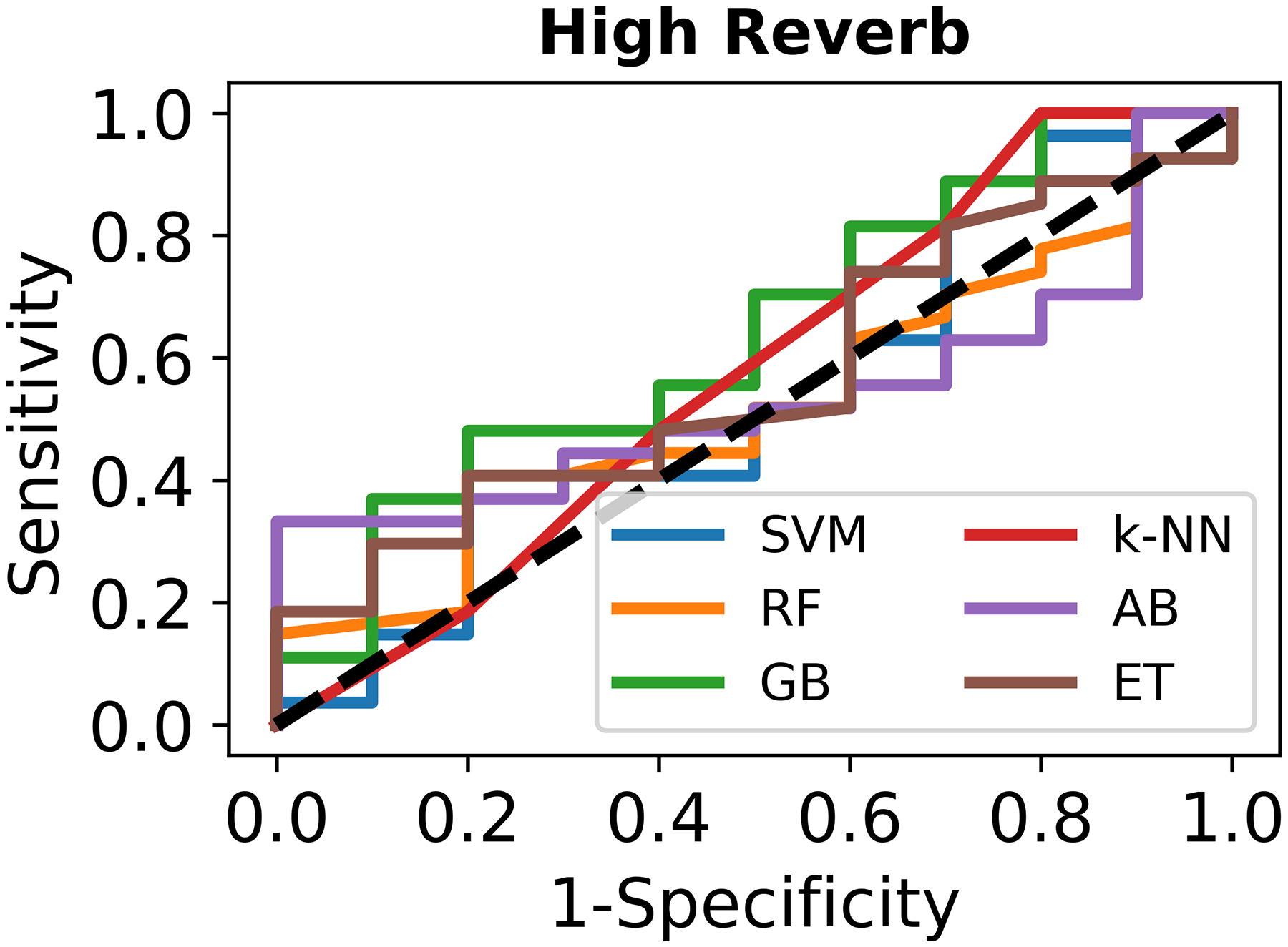

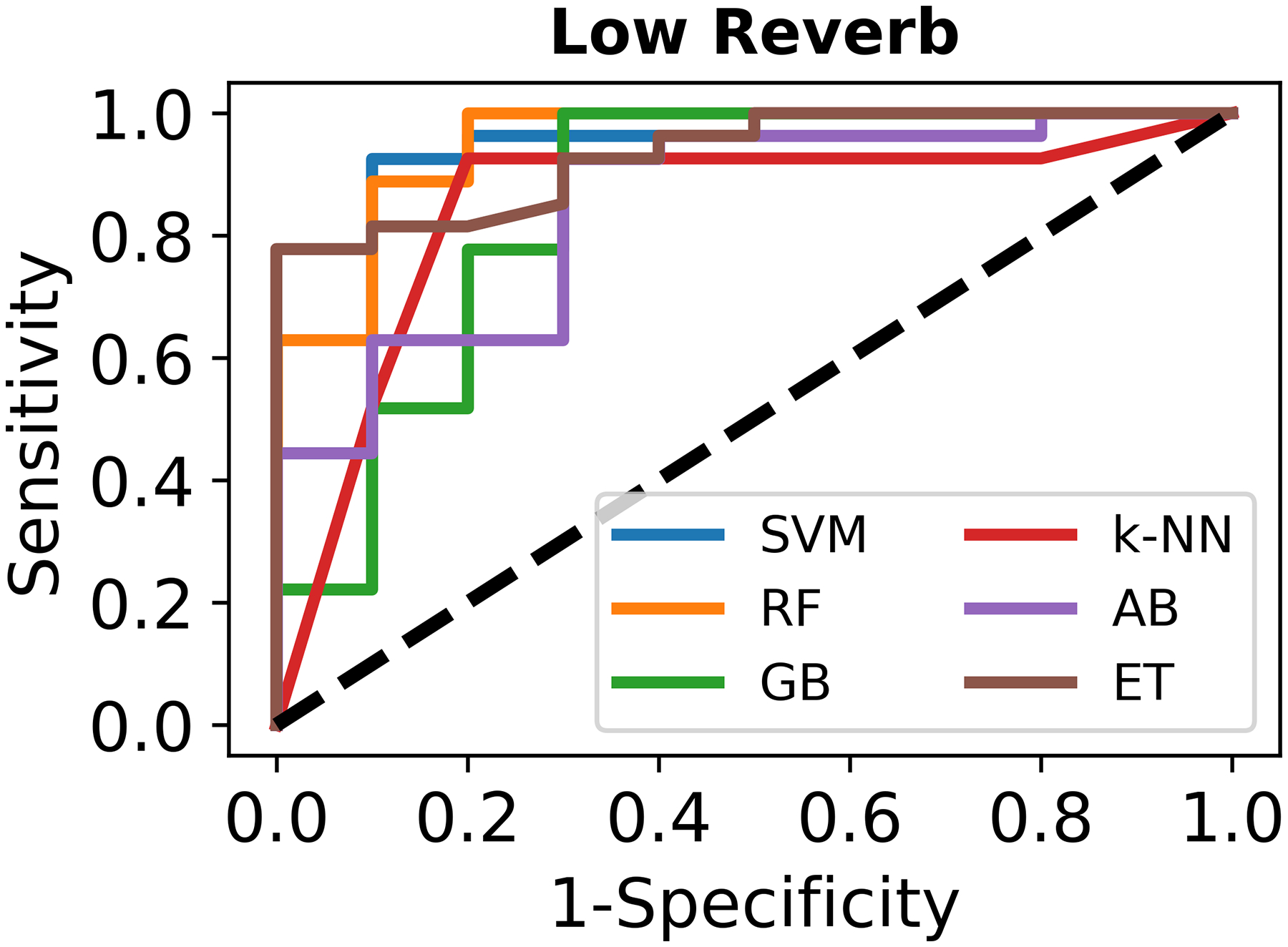

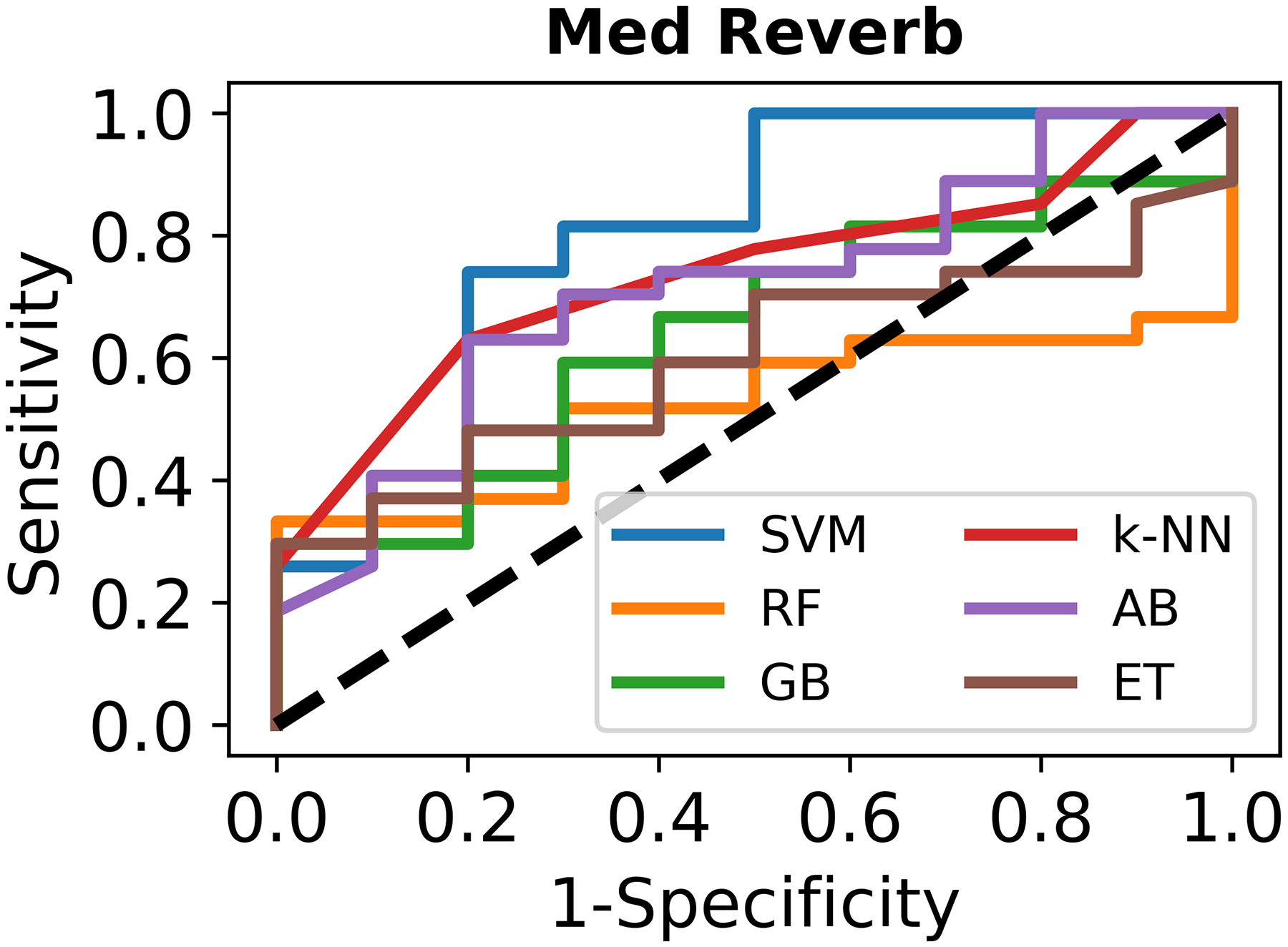
Receiver operating characteristic curves to evaluate the performance of the machine learning classifiers: (**a**) Testing on the original testing recordings set with low reverberation; (**b**) Testing on the original testing recordings but mixed with medium reverberation effect (reverberation time = 0.48 s); (**c**) Testing on the original testing recordings but mixed with high reverb effect (reverberation time = 1.82 s). The models include Random Forest (RF), Gradient Boosting (GB), Support Vector Machine (SVM), Extra Trees (ET), AdaBoost (AB), and k-Nearest Neighbors (k-NN).

The results compare the ML models across different accuracy metrics under low, medium, and high reverberation, testing their sensitivity to simulated recording conditions. Figure 1 illustrates ROC curves for ML classifiers evaluated on three testing datasets with different challenging conditions (low, medium, and high reverberation levels added on the testing recording). Sensitivity is plotted against 1-specificity. In the low-reverberation dataset (a), most models demonstrate high sensitivity and specificity, with steeper ROC curves approaching the upper left corner. In the med-reverberation dataset (b), the performance of all the classifiers declines moderately, reflected in flatter ROC curves compared to the low-reverberation condition. SVM and k-NN showed relatively better sensitivity and specificity among the other models, while RF exhibited a noticeable drop in performance. Under high reverberation (c), performance of all models further diminishes, with ROC curves approaching the diagonal line (indicating random performance with no discriminatory ability).

**Figure 2.**
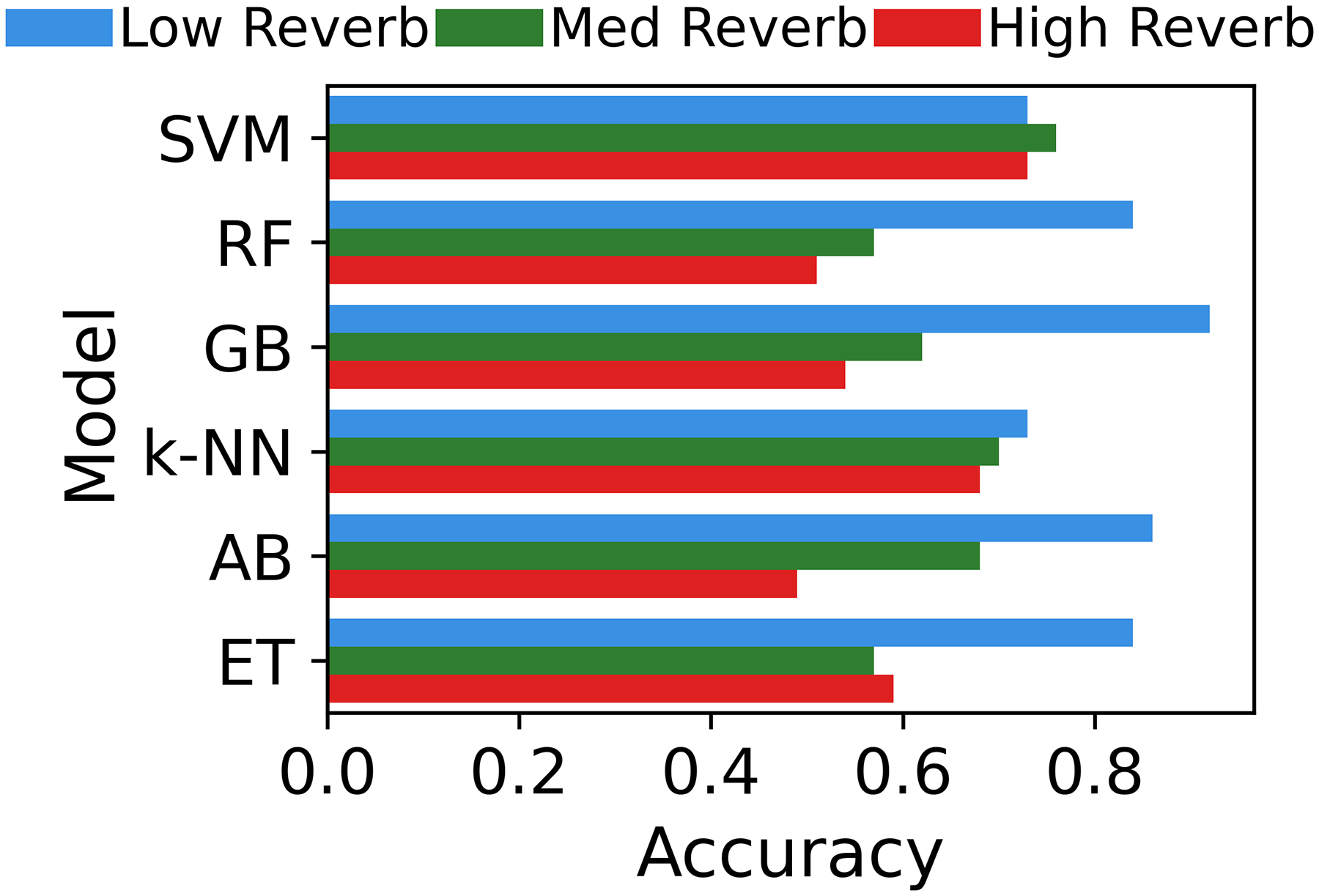

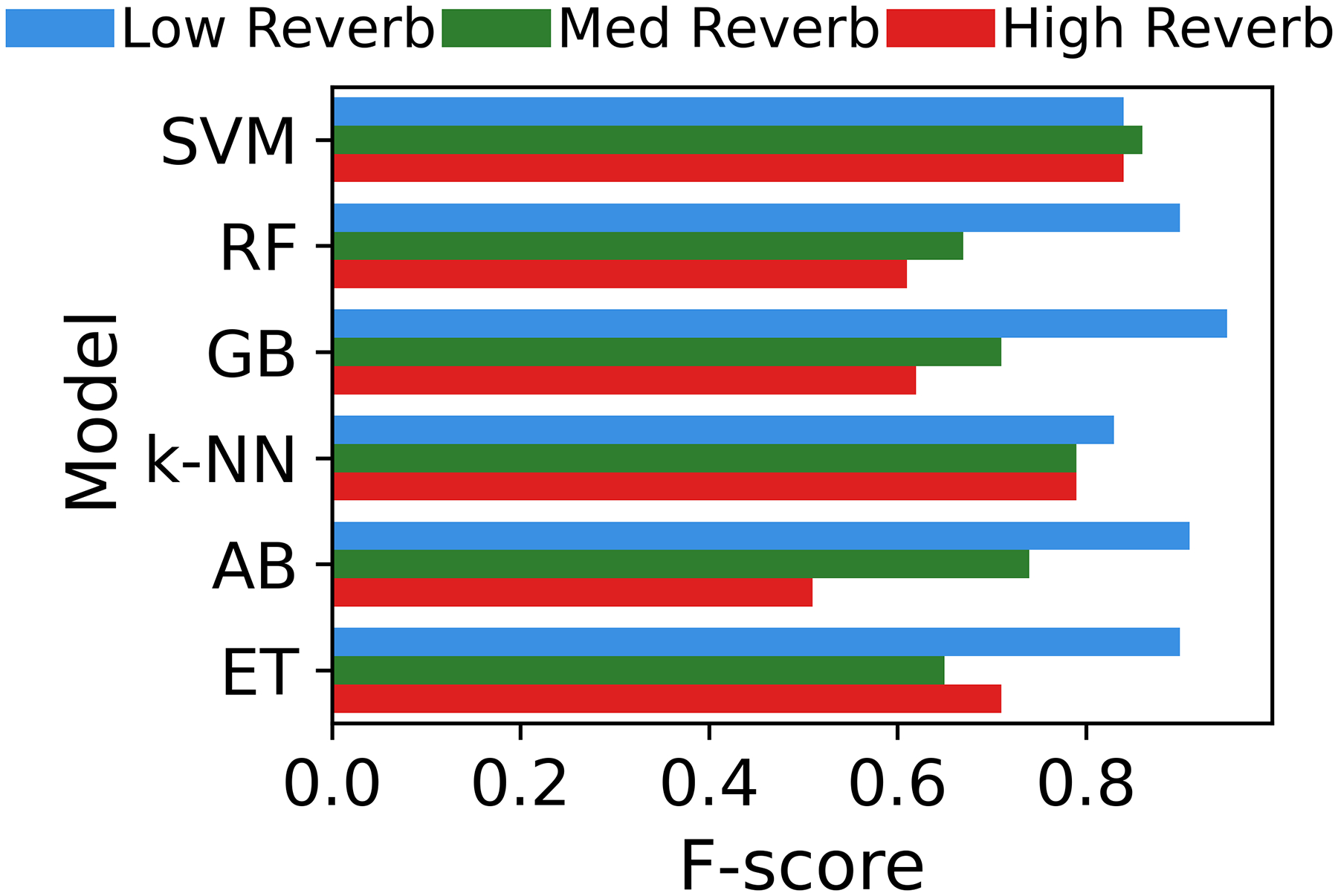

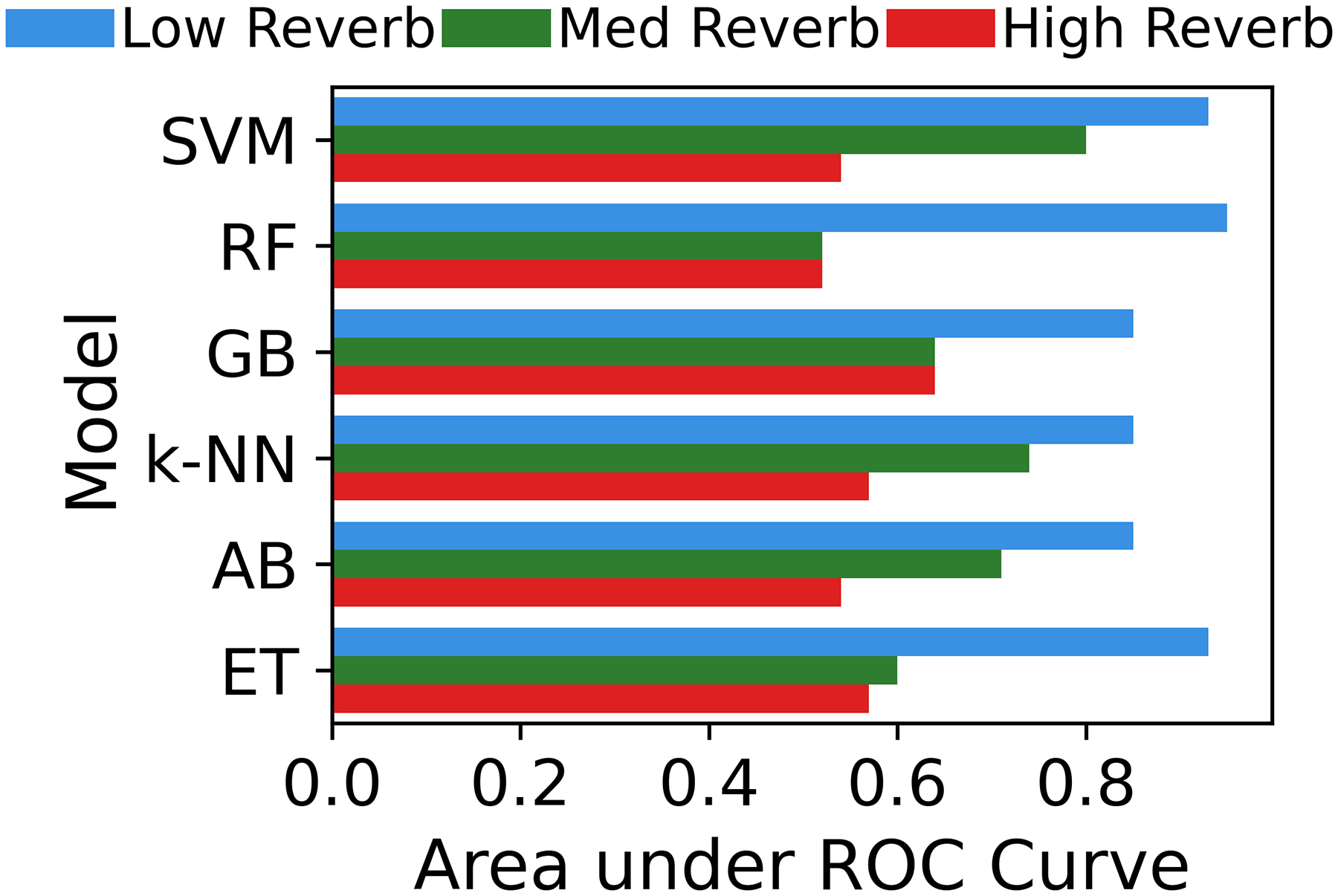
(**a**) Accuracy; (**b**) F-score; (**c**) Area under receiver operating characteristic (ROC) curves to test the machine learning classifiers under three conditions: on the original testing recordings set with low reverberation (light blue); on the original testing recordings but mixed with medium reverberation effect (reverberation time = 0.48 s) (in green); on the original testing recordings but mixed with high reverberation effect (reverberation time = 1.82 s) (in red). The models include Random Forest (RF), Gradient Boosting (GB), Support Vector Machine (SVM), Extra Trees (ET), AdaBoost (AB), and k-Nearest Neighbors (k-NN).

The performance metrics—accuracy (a), F-score (b), and AUC (c)—of the classifiers are plotted in Figure 2 across the three testing datasets. SVM and k-NN maintained relatively high scores across all metrics, with almost no changes in accuracy and F-score across the three conditions. The figure, in contrast, shows that RF achieved the highest scores across all metrics in the low-reverberation condition, though these scores dropped noticeably, ranking among the lowest under the med- and high-reverberations. The rest of the models also exhibit a clear decline in their accuracies, but at varying levels of impact.

## 4. Discussion

This paper presents a comparative evaluation of various ML models for classifying voice samples as nonpathological or pathological. While previous studies have explored the potential of ML in detecting voice pathology from audio recordings with promising results [1,2,4], this work takes an additional step by testing the generalizability and robustness of common ML models. By augmenting the testing dataset with med- and high-reverberation conditions, creating more challenging versions of the recordings, this study identifies the most robust models that are less sensitive to variations in recording quality during data collection.

Overall, the results highlight the varying robustness of ML models under simulated recording conditions (reverberation levels), emphasizing the impact of environmental factors on the performance of audio-based ML models. SVM demonstrated consistent robustness, making it a strong candidate for real-world voice assessment applications with variable recording conditions. Similarly, k-NN, while not a top performer on clean recordings, was less affected by reverberation and ranked among the best under adverse conditions. In contrast, RF revealed the highest classification accuracy on the clean recordings, aligning with its strong performance reported in the literature [1,31], but its accuracy dropped significantly with augmented recordings, revealing high sensitivity to environmental variability and limited generalizability. These findings underscore the importance of evaluating models under challenging and diverse testing conditions, as high accuracy on clean datasets alone can be misleading. Real-world robustness requires testing with varied techniques and data collection environments to ensure reliable performance.

## 5. Conclusions

This study underscores the importance of testing machine learning models for voice disorder classification under diverse recording conditions (data quality), an important step towards more widespread usability of ML models. Various ML models were evaluated on augmented recordings with simulated reverberation to mimic adverse acoustic environments and audio data quality. SVM and k-NN demonstrated consistent reliability under these conditions, whereas RF, despite strong performance on low reverberation conditions, showed reduced generalizability with heavily augmented recordings with higher reverberation. These findings highlight the necessity of training and evaluating models on datasets that reflect real-world acoustic environments to ensure reliable and practical applications in voice assessment.

## Data Availability

The data presented in this study are available on request from the corresponding author. The data are not publicly available due to ethical restrictions.
